# The effects of educational program on health volunteers’ knowledge regarding their approach to earthquake in health centers in Tehran

**Published:** 2015-04

**Authors:** ZAHRA JOUHARI, AFSHAR PIRASTEH, GHOLAM REZA GHASSEMI, LEILA BAZRAFKAN

**Affiliations:** 1 Faculty of Medicine, Shahed Universit, Tehran, Iran;; 2 Medical Education Center, Isfahan University of Medical Sciences, Isfahan, Iran;; 3 Quality Improvement in Clinical Education Research Center, Shiraz University of Medical Sciences, Shiraz, Iran;

**Keywords:** Health, Education, Earthquake

## Abstract

**Introduction:**

The people's mental, intellectual and physical non-readiness to confront earthquake may result in disastrous outcomes. This research aimed to study of effects of a training intervention on health connector’s knowledge regarding their approach to earthquake in health-training centers in East of Tehran.

**Methods:**

This research which is a semi-experimental study was designed and executed in 2011, using a questionnaire with items based on the information of Crisis Management Org. After a pilot study and making the questionnaire valid and reliable, we determined the sample size. Then, the questionnaires were completed before and after the training program by 82 health connectors at health-treatment centers in the East of Tehran. Finally, the collected data were analyzed by SPSS 14, using paired sample t–test and Pearson's correlation coefficient.

**Results:**

Health connectors were women with the mean age of 43.43±8.51 years. In this research, the mean score of connectors’ knowledge before and after the training was 35.15±4.3 and 43.73±2.91 out of 48, respectively. The difference was statistically significant (p=0.001). The classes were the most important source of information for the health connectors.

**Conclusion:**

The people's knowledge to confront earthquake can be increased by holding training courses and workshops. Such training courses and workshops have an important role in data transfer and readiness of health connectors**.**

## Introduction

Floods, storms, and earthquakes are the incidents which always affect the residents in the affected region. Earthquakes are devastating, particularly in developing countries, because of the usually high populations and generally poor building standards (1). Although disasters are unpredictable as to when and where they strike, the resulting damage often paralyzes local government structures and systems (2).

Iran as one of the earthquake-prone countries of the world has experienced 18 earthquakes with more than 7 Richter which have been led to a lot of physical and financial damages. Iran holds the 6^th^ rank in the world regarding natural disasters (3). Research has shown that earthquake causes a lot of physical, mental and social damage, a remarkable part of which is preventable (4).

In this research we attempted to investigate the effects of a training intervention on the Health connectors of Tehran health-treatment centers regarding their readiness to confront earthquake before, while and after its occurrence and the increase of their knowledge on first aids in line with relief in case of fracture, bleeding and heart arrest. Increasing the health connectors’ readiness leads to data transfer to their families and other people. Peate in his research in Arizona suggested readiness training for disaster confrontation for tribal leaders and considers this method as the way for data transfer to other groups of people (5, 6). Fuady also considers the potentials of health and training centers’ volunteers essential to earthquake damage reduction (4).

The main cause of losses, damages and disastrous outcomes of earthquakes in Iran and other countries is non-standard buildings, and mental, intellectual and physical unreadiness of people to confront earthquake and immobilization of unpredictable accidents management system and relief forces and rehabilitation of the people (stratum). These factors lead to the following outcomes.

1. Expansive destruction results in high human losses and financial damages.
2. Delay in accurate identification of earthquake location results in waste of golden hours of relief and increase of secondary wastes.
3. People's non familiarity with self-aid operation and lack of sufficient facilities result in death of a large number of injured people before relief forces' arrival. Due to people's non familiarity with shelter seeking during earthquake and safety actions, the mortality rate will be increased.

Rashidi in a study in 2012 in Tehran showed that Tehran is very susceptible to earthquakes and according to the law of reversibility and repetition of natural disasters, the probability of earthquakes is very high and the critical areas are precisely occupied by the lower strata of the society (7).

Shokouh in a study in 2012 in Tehran showed that 86.7% of hospitals were in good preparedness level against earthquake (8). However, it is necessary for people to be prepared against earthquake. This identifies a need for more effective earthquake education programs. It is important to understand how people make sense of hazards and make decision about how to manage the associated risk (9).

Schools can play a crucial role concerning training and building a disaster prevention culture, among various community groups. Principals and teachers have a key role to play in any school-wide initiative through developing and reviewing awareness policy, developing and revising emergency response plans, holding emergency drills and training the students (10).

Researches done in other countries confirm that for damage decrease, training employees and other people and registering the objects of disasters in medical training systems should be obligatory (11-13).

**Method of Earthquake Confrontation before Occurrence: **Planning to confront earthquake outcomes and inform the public may decrease harms and damages of earthquake to properties and physical damages to the people. Readiness includes all actions and policies done before occurrence of unpredictable accident for prevention. Readiness includes practice actions and maneuvers and planning for actions after accident (8).

**Method of Earthquake Confrontation at the time of Occurrence: **The trained people know how to analyze a situation. They think positively and flexibly and show proper behavior. Regarding the Health connectors’ role, some specific trainings should be considered for this stratum so that they protect themselves against damages on the one hand and help others with lowest losses on the other hand. These trainings should be repeated in time periods, if necessary, in order that information is stabilized (14, 15). Trainings related to the time of earthquake occurrence includes trainings in the field of quality of escape from dangerous situations, method to rescue injured people, methods to provide first aids, methods for prevention of fire and extinguishing it, environment renovation methods, prevention of infectious diseases at the earthquake area and methods to supply water in emergency situations. Basic trainings in this phase will be so effective and non-readiness and no training in this phase will make many problems and losses (16-18).

**Method of Earthquake Confrontation after Occurrence:** Providence and planning for change of this status into better status in advanced countries result in human damages and incurrence of social-economic costs in case of occurrence of natural disasters. Nonexistence of these two features i.e. providence and planning result in death of thousands of innocent people against disasters such as earthquake in countries like Iran.

If the previous plans are made in a proper way in the after-earthquake event, just following the plans would be enough. The most important pints after the crisis, are locating damaged areas, saving humans lives and providing food and accommodation. His proves that to keep the earthquake stricken people alive. Skilled forces are required to rescue and first aids (19).

## Methods

This study is a kind of semi-experimental research which was done on a before-after basis in Feb. 2011. The population included health connectors who referred to East of Tehran Health-Treatment Centers. This group permanently and voluntarily communicates with centers and transfers training to other people in their neighborhood. They were selected for this purpose due to the limitations and the necessary involvement of the East of Tehran Health-Treatment Centers covered by Shahid Beheshti University.

A questionnaire was designed after reviewing the literature, the existing references at Red Crescent and Crisis Management Organization and need analysis of the health connectors. This questionnaire contained demographic particulars, experience of earthquake confrontation, first aids training, information sources, training record of earthquake confrontation and questions related to the knowledge of approaching earthquakes (16 questions related to actions before earthquake, 16 questions related to actions at the time of earthquake and 16 questions related to actions after earthquake). Correct answers were scored 1, and wrong and I don’t know answers were scored 0. After primary drawing up and inspecting for financing, the questionnaire was sent to several specialists of Health Dept and based on their comments, it was revised and validated. The primary questionnaire was tested with 10 health connectors in a pilot study. For reliability, Test-Retest was used after 1 week (r=0.7). Then, the final questionnaire was drawn up and the sample population was determined based on statistical formulas, which was equal to 80 people.

This sample size was available regarding number of Health connectors at any center, at 5 centers (17^th^ Shahrivar, Zarenejad, Homayoon, Samangan and Afsareyeh) and training started by the participation of all Health connectors. This study tested the hypothesis that training increases health connector’s knowledge in their approach to earthquake.

After coordinating with Health-Treatment Centers Supervisors and obtaining permission, the training project was prepared for Health connectors. First, in a session, the significance of subject was explained to the Health connectors, then a questionnaire concerning readiness for earthquake confrontation was given to the Health connectors to complete before being trained. Thereafter, the subjects were educated in the form of lecture and group discussion, using training posters at a training workshop. After completion of the training session and a short rest time, the post-test stage was done. Finally, the training subjects in the form of pamphlet, book and CD were gifted to the Health connectors. 

The ccompleted questionnaires were coded for data analysis. Then the data was registered to SPSS 14. After determining normal readiness score and other quantity information, standard deviation and frequency were used for statistical data analysis. Comparison of the means before and after the study was done with Paired sample t –test and Pearson's correlation coefficient were used for the comparison of correlations. The ssignificance level in this study was 0.05. 

## Results

Health connectors completed 164 questionnaires (82 pre-test questionnaires and 82 post-test questionnaires) in health centers (17^th^ Shahrivar, Zarenejad, Homayoon, Samangan and Afsareyeh). The mean of health connector's age was 43.43±8.51 and all were women. The maximum age was 51.94 and the minimum age was 34.92. 9.8% of health connectors had Elementary School degree, 22.0% were ^3rd^ grade of guidance school, 53.6% had high school diploma and 14.6% had associate degree and higher. 57.3% of Health connectors had completed first aids training, seven percent of them at health centers, thirty six percent at Red Crescent Org, thirty four percent at other centers and thirteen percent were trained at centers on integration basis. 42.7% of Health connectors had completed training course on earthquake confrontation. 25.7% had completed the training course of earthquake confrontation at Health-Treatment Centers, 17.1% at Red Crescent Org., 45.7 % at other centers, and 11.5% had completed training course of confrontation earthquake at centers on integration basis.

Health connectors had Red Crescent Relief and Rescue Bag at home (13.4%). Over seventy percent (72.7%) of the group having Relief and Rescue Bag had completed first aids training. In addition, 45.5% of the group having Relief and Rescue Bag at home, had completed training courses of earthquake confrontation. The health connectors had confronted earthquake in the last one year (29.3%). 

The Health connectors declared that the most proper information source in earthquake confrontation was the training courses held at centers, TV, radio, magazines, newspapers, relatives and friends (Table 1). We compared health connectors’ knowledge score in different health centers before and after training (Table 2).

**Table 1:** Mean, standard deviation and priority of information sources based on the opinion of East of Tehran Health-Treatment Centers Health connectors

Information source	Mean±SD	Min	Max
Radio	2.78±0.96	1	5
TV	4.33±0.50	3	5
Magazines	2.07±0.68	1	4
Class	4.41±0.83	2	5
Friends	1.12±0.45	1	4

**Table 2:** Mean score of awareness of East of Tehran Health-Treatment Centers Health connectors before and after training at each center

Center	Before education	After education	p
	Mean±SD	Mean± SD	
17 Sharivar	34.17±3.68	42.50±2.55	0.001
Zarenejad	36.20±5.38	42.73±2.91	0.001
Homayoon	36.85±4.52	41.64±3.10	0.001
Samagan	37.05±3.84	45.30±1.49	0.001
Afsareyeh	36.60±4.13	46.07±1.98	0.001

* General score has been 48

The mean score of Health connectors before and after the training was 36.15±4.3 and 4.73 out of 48 (Table 3). The difference between their knowledge before and after the training was statistically significant (p= 0.001).

**Table 3:** Mean and standard deviation of awareness score of health connectors before and after training

	Before education	After education	p
	Mean± SD	Mean± SD	
Before earthquake	12.93±1.48	15.18±1.02	0.001
When earthquake	12.02±1.83	14.84±1.08	0.001
After the earthquake	11.18±2.18	13.17±1.41	0.001
Total	36.15±4.33	43.73±2.91	0.001

* General score: 48

There was a significant inverse relationship between age and increase of knowledge score before training (r=-0.27, p=0.012). Before training, there was a significant difference between knowledge score of those who had passed first aids training and those who had not passed it (p= 0.001). Averages of awareness score for those trained in first aids and for those not trained were 37.45±4.31 and 3.74±34.40, respectively. There was a significance relationship between record of earthquake confrontation and awareness (p=0.045). The Chi–square test showed that there was a significant relationship between providing relief bag and first aids training (p=0.001). 

## Discussion

In this research, the awareness score increased an educational workshop. In a research done by Mahalati and Firooz in 2004, the results showed that more public education resulted in more readiness to confront earthquake. Tekeli’s research showed that higher educational levels and social factors can be the effective factors in readiness (20, 21).

This research showed that participation in training courses at Centers and on TV can transfer information to the Health connectors before training. After training courses, TV was the audiovisual medium which may play a remarkable role in public training. Hunng in his research in China found that people prefer informal training. Therefore, despite personal training advantages, the training should be considered through other resources (22). Rajib in his research in China found that schools played the most key roles in information transfer. The significance of family, society and self-training should be emphasized. Since this research was done on the students, the school had the most roles in training for transfer of information (23).

Since all Health connectors participating in this research were women, we cannot comment on the role people's sex. Research done by Falahi and Parsizadeh in 1996 in Ardebil earthquake stricken area showed that sex was effective in the first step at the time of earthquake, but was not effective in relieving after earthquake (24). Ghaffari Nejad in his research in Bam showed that sadness resulting from earthquake was more in women having low training level. This is so important regarding their role in family (25).

This research did not show meaningful relationship between earthquake and awareness score. Since in recent years no destructive earthquake has occurred in Tehran and they have not performed any serious action in this respect, therefore this result is predictable. Rajib in his research in China showed the same result (22).

This research showed that there was a significant relation between first aids training and awareness score. This research showed that the people's awareness of the earthquake should be increased by training. The research done by Francisco Horton in New Zealand in 1997 showed that there was a meaningful relationship between earthquake awareness and people’s readiness against it (26). Ellidokuz in his research in Turkey in 2002 observed that the most important risk factors in morality and losses resulting from earthquake were destruction state of the earthquake and the place where (insecure place) the person was at the time of earthquake (27). Therefore, identification of secure places should be effective in decreasing the effects of destruction.

In a research done by Paton Douglas in Australia in 2006, the role of training for danger confrontation, adaptability and decrease of risks in the society and people was emphasized. These trainings included quality of structure and installations engineering and people and society's readiness against natural disasters (28). Necessary readiness in all governmental and nongovernmental sections for disaster confrontation is so important. In a research done by Rumoro in the USA in 2010 for trial assessment of the emergency physicians’ readiness in disaster confrontation showed that emergency physicians did not have the required readiness (29). Training and identification of shelter seeking at the time of earthquake extremely decreases physical losses and injury to people. The research done by Wen in South West of China in 2009 showed that the main factor of mortality was strong strokes and trauma on head and other factors such as infection. This research showed that identification of secure places and shelter seeking status of the people was so important (30). Raissi in his research on Bam Earthquake showed that an important priority in training is bleeding (31). Strength of buildings is an important factor in protection of the people's life. A research done by Ardalan in 2006 under the title of factors affecting mortality, losses in direct relation with earthquake showed that destruction of building affects mortality a lot. Therefore, construction strengthening against earthquake has high priority. However, operational strategy predetermined by State organizations for the decrease of physical and financial losses is obligatory (32).

Beeker in his qualitative study showed that how beliefs and competencies at personal, social, and environmental levels interact to influence people**’**s risk management choise. Three main categories of beliefs were found previously to influence the preparedness and personal beliefs (33).

## Conclusion

The society's readiness in all aspects for earthquake and critical situations should be obligatory. Promotion of the society's readiness to confront the future crises requires comprehensive planning. Training the groups who help others should be considered important.

It is recommended that future studies notice other key people that may be useful in preparedness against earthquake. Education can provide useful information for such cases. In school education, more active ways of disaster education through experience, and visual aids are found to be effective.

**Limitation:**

This study had some potential limitations that may affect the results. The study was limited to health centers of a single university. Therefore, generalizability of results can be considered as a limitation of the present study. An insufficient literature, particularly at the international level is another limitation of this study. The best important strengths in this study are related to the interesting topic and special participants. 
